# Validation of the French version of the McLean screening instrument for borderline personality disorder (MSI-BPD) in an adolescent sample

**DOI:** 10.1186/s12888-020-02643-8

**Published:** 2020-05-12

**Authors:** Bojan Mirkovic, Mario Speranza, Lionel Cailhol, Julien-Daniel Guelfi, Fernando Perez-Diaz, Maurice Corcos, Marion Robin, Alexandra Pham-Scottez

**Affiliations:** 1 Service de Psychiatrie de l’Enfant et de l’Adolescent, CHU Charles-Nicolle/ CH Le Rouvray, Normandie Université, 1 rue de Germont, 76000 Rouen, France; 2 Université Paris-Saclay, UVSQ, INSERM, Centre for Research in Epidemiology and Population Health (CESP), Team “DevPsy”, 94807 Villejuif, France; 3 Service Universitaire de Psychiatrie de l’Enfant et de l’Adolescent, Centre Hospitalier de Versailles, Versailles, France; 4 CIUSSS de l’Est-de-l’Île-de-Montréal, Université de Montréal, Québec, Canada; 5 GHU Paris Psychiatrie et Neurosciences, Paris, France; 6 CNRS–UPSR 3246, Hôpital de la Salpêtrière, Paris, France; 7 Département de Psychiatrie de l’Adolescent et du Jeune Adulte, Institut Mutualiste Montsouris, Paris, France; 8 Université Paris Descartes, Paris, France

**Keywords:** Borderline personality disorder, Adolescent, Screening, MSI-BPD

## Abstract

**Background:**

The study examines the psychometric properties of the French version of the McLean Screening Instrument for Borderline Personality Disorder (MSI-BPD) created by M. Zanarini to screen borderline personality disorder in clinical and non-clinical populations.

**Method:**

In this multicentric longitudinal study from the European Network on Borderline Personality Disorder, a sample of 84 adolescent patients from five psychiatric centres and 85 matched controls without psychiatric comorbidity completed the MSI-BPD, French version, and were interviewed with the Structured Interview for DSM-IV Personality (SIDP-IV), in order to assess the presence or absence of borderline personality disorder.

**Results:**

The MSI-BPD showed excellent internal consistency (α = 0.87 [0.84;0.90]). Compared to the semi-structured reference interview (SIDP-IV), the MSI-BPD showed substantial congruent validity (AUC = 0.93, CI 95%: 0.90–0.97). The optimal cut-off point in the present study was 5 or more, as it had relatively high sensitivity (0.87) and specificity (0.85). In our sample, the cut-off point (7 or more) proposed by the original developers of the MSI-BPD showed high specificity (0.95) but low sensitivity (0.63).

**Conclusions:**

The French version of the MSI-BPD is now available, and its psychometric properties are satisfactory. The French version of the MSI-PBD can be used as a screening tool for borderline personality disorder, for clinical purposes or in research studies.

## Background

Borderline personality disorder (BPD) is frequent, about 1–2% in general population [1]. Iit is also the most frequent personality disorder among psychiatric samples of in- and out-patients [2] and is largely under-diagnosed in clinical practice [3]. BPD is associated with severe chronic impairment, and repetition of suicide attempts and / or self-injurious behaviours, as well as other forms of impulsive behaviours, and requires high levels of medical and psychiatric treatment services [4]. The frequent presence of psychiatric comorbidities makes the diagnosis of BPD more difficult [5]. However, an early detection of BPD would allow for better patient care and reduce health costs, since specific psychotherapies (such as the Good Psychiatric Management or Dialectical Behavior Therapy and Mentalisation-Based Therapy) have demonstrated their effectiveness [6]. Several authors have recommended a two-stage BPD diagnostic approach [7]. The first step is to use a fast, reliable screening instrument and then, in the event of positive results, a semi-structured interview (such as the DIPD or the SIDP-IV), the usual gold standard diagnostic procedure for BPD [8]. In this context, in 2003, Zanarini and colleagues [5] developed a brief instrument to screen for BPD, the McLean Screening Instrument for Borderline Personality Disorder (MSI-BPD), which is derived from the BPD module of the Diagnostic Interview for DSM-IV Personality Disorders [9]. It is a yes/no, self-report questionnaire of ten items, one for each DSM-IV-TR [10] BPD criterion, except two items for the paranoia/dissociation criterion. In the validation study, the MSI-BPD demonstrated good psychometric properties, with both high sensitivity (0.81) and high specificity (0.85), with a cut-off score of 7 or more items. Diagnostic efficiency was even higher (with the same cut-off) when subjects were younger: sensitivity = 0.87 and specificity = 0.90 for people younger than thirty, sensitivity = 0.90 and specificity = 0.93 for people younger than twenty-five. Based on this latter finding, the authors suggested that the MSI-BPD may be ideal as a screening instrument in late adolescent and young adult samples.

The recent literature review by Zimmerman and Balling [11] reports 12 studies of the MSI-BPD, including 3 studies in adolescent and young adult populations [12–14]. The sensitivity of the scale ranged from 48.4% [12] to 91% [15] and the specificity of the scale ranged from 19.2% [15] to 90.2% [16]. A maximization approach to select an optimal cut-off (the best balance between sensitivity and specificity) was widely used and the authors recommended cut-offs ranging from 5 [12] to 8 [13]. It should be noted that 5 authors recommended a threshold of 7 [5, 15, 17–19].

The goal of our study was to explore the psychometric properties of the French version of the MSI-BPD.

## Methods

### Participants and procedure

First, we translated the MSI-BPD into French, with the agreement of the developer of the instrument, Pr. M. Zanarini. The French MSI-BPD was then back-translated, and conformity with the original version was verified. Some members of our team also coordinated the translation of the DSM-IV [10] and the DSM-IV-TR [20], so we were familiar with the English-French translation issues.

This study is part of a larger multicentric longitudinal study of BPD in adolescence from the European Research Network on BPD, described in detail elsewhere [21]. The research network was composed of 5 academic psychiatric centres in France, Belgium, and Switzerland. During the period from January to December 2007, all in- and out-patient adolescents (15 to 19 years old) were clinically screened by the consulting psychiatrists following the DSM-IV criteria for borderline personality disorders. Adolescents fulfilling a clinical diagnosis of BPD were referred to the research team for further assessment. The exclusion criteria were: mental retardation, schizophrenic disorder, serious mental illness, pregnancy, refusal to participate, and inability to understand French. 107 patients with a possible diagnosis of BPD were referred to the study by their clinicians. Of these subjects, 84 fulfilled SIDP-IV criteria for a BPD. Among the adolescents included, 67% (*N* = 56) were inpatients. Concerning psychiatric comorbidities at inclusion, 37% (*N* = 31) had a major depressive disorder, 35% (*N* = 29) had an eating disorder, 26% (*N* = 22) had an anxiety disorder, and 20% (*N* = 17) had a substance abuse disorder.

The control sample included 85 healthy adolescents matched for age, gender and socio-economic status. Control subjects had to be exempt of BPD, and to have no lifetime follow-up with a psychiatrist or a psychologist.

A detailed explanation of the study goals and procedures was given to the participants. Each patient or subject who agreed to participate signed a written consent form. Written informed consent was also obtained from at least one parent, if the participant was younger than 18 years old. This study was approved by the relevant French ethical committees (‘Comité Consultatif sur le Traitement de l’Information en matière de Recherche dans le domaine de la Santé’ – CCTIRS, and ‘Comité de Protection des Personnes’ – CPP), and all the results were collected in an anonymous database, approved by the French Data Protection Authority (Commission Nationale Informatique et Libertés - CNIL).

### Measures

All the participants completed the French translation of the MSI-BPD, and were investigated with the Structured Interview for DSM-IV Personality [8] by interviewers who were blind to their MSI-BPD responses and their patient/control status. The SIDP-IV is a standardized semi-structured interview that assesses each of the ten DSM-IV personality disorders (including BPD). The research team was composed of five interviewers (psychologists and psychiatrists), all familiar with the standardized instruments and trained for this study. Inter-rater reliability for the SIDP-IV was calculated from independent ratings of ten videotaped interviews. The Kappa coefficient for the presence/absence of a BPD was very good = 0.84. The intraclass correlation coefficient for the borderline SIDP-IV score was excellent = 0.95.

### Statistical analyses

We first ran univariate comparisons of sociodemographic variables between BPD and non-BPD subjects. Our main analyses aimed to assess the MSI-BPD scale validity in French. First, a factor analysis using varimax rotation was performed on the ten scale items using the tetrachoric correlations matrix as input. The optimal number of factors was chosen by visual inspection of the scree plot with Package “psych” - R [22]. We then checked the concurrent validity of the MSI-BPD with the SIDP IV using Spearman’s rank correlations. We computed a ROC curve to find a cut-off threshold ensuring optimal sensitivity and specificity, according to the Youden’s statistic. Finally, reliability assessment was done using Cronbach’s alpha statistic (the psych package). The analyses were run on R 3.5.1, and a *p*-value less than 0.05 was considered significant [23].

## Results

84 patients diagnosed as having a BPD according to the SIDP-IV participated in the study. The mean age of patients was 17 [16, 17], 86% (*n* = 73) were female, and 91% were high school students. 88 controls participated in the assessments, with 3 controls being excluded because of prior psychological or psychiatric consultations. The mean age of the control group was 16 [15–17], 76% (*n* = 64) were female, and all were high school level. There was no significant difference between the two groups concerning age, gender and level of education, respectively *p* = 0.08, *p* = 0.073 and *p* = 0.35.

### The factor structure and internal consistency of the MSI-BPD

**Fig. 1:**
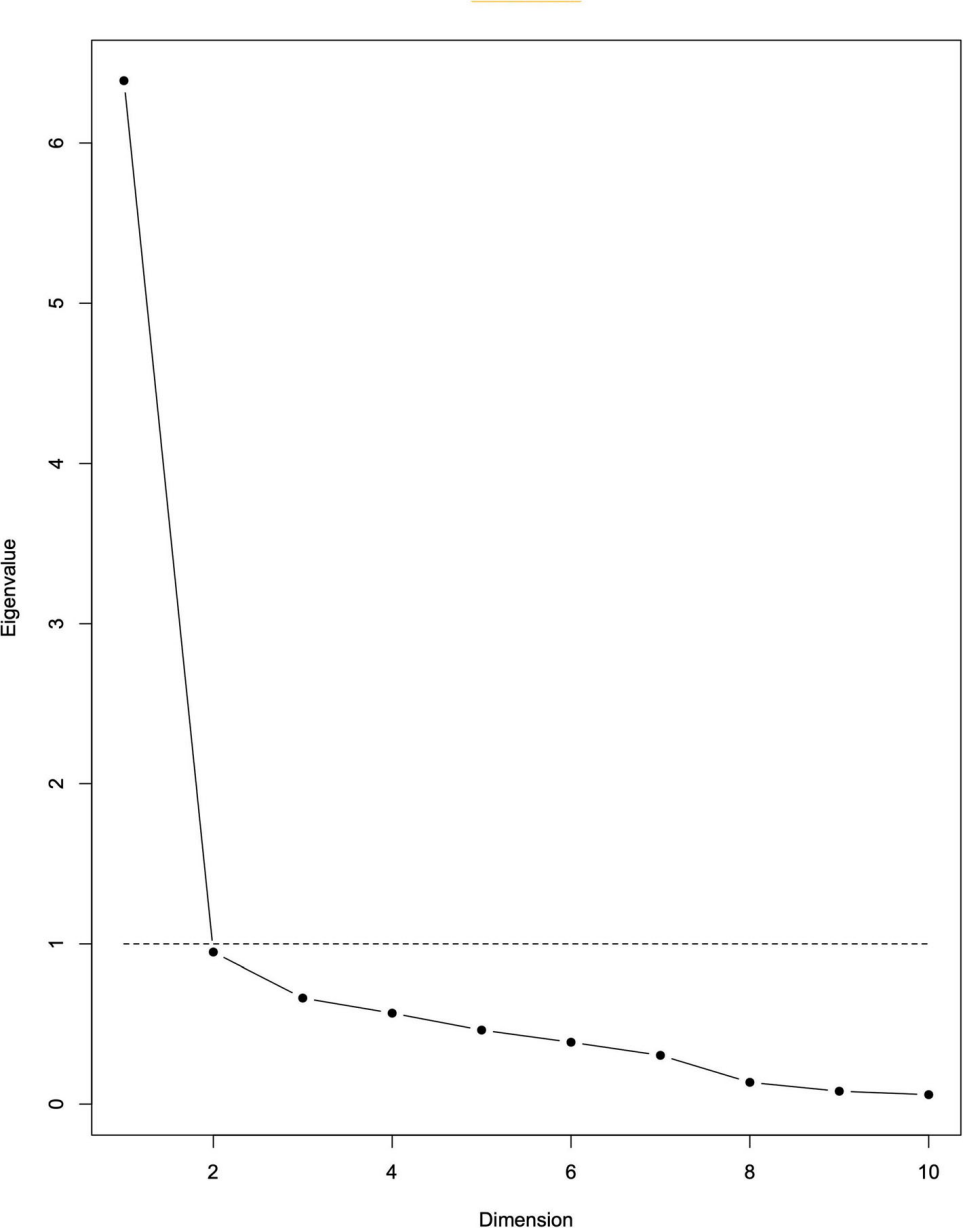
Sedimentation graph of factor components of 10-item MSI-BPD (Scree Plot)

Exploratory factor analysis indicated one component that explained 60.1% of the variance (Table 1). The scree plot was used to establish the number of distinct factors (Fig. 1). Results show one dominant dimension with a distinct angle between the first and second components, suggesting the unidimensionality of the MSI-BPD. The internal consistency for the MSI-BPD scale was excellent (alpha = 0.87 [0.84;0.90]).

**Table 1:** One factor solution and item factor loadings for the MSI-BPD

Item number and content	Component
	**Factor 1**
Q1-Have any of your closest relationships been troubled by a lot of arguments or repeated breakups?	0.77
Q2-Have you deliberately hurt yourself physically (e.g. punched yourself, cut yourself, burned yourself)?	0.87
How about made a suicide attempt?	
Q3-Have you had at least two other problems with impulsivity	0.79
(e.g. eating binges and spending sprees, drinking too much and verbal outburst)?	
Q4-Have you been extremely moody?	0.85
Q5-Have you felt very angry a lot of the time? How about often acted in an angry or sarcastic manner?	0.72
Q6-Have you often been distrustful of the other people?	0.72
Q7-Have you frequently felt unreal or as if things around you were unreal?	0.67
Q8-Have you chronically felt empty?	0.91
Q9-Have you often felt that you had no idea of who you are or that you have no identity?	0.74
Q10-Have you made desperate efforts to avoid feeling abandoned or being abandoned (e.g. repeatedly called someone to reassure yourself that he or she still cared, begged them not to leave you, clung to them physically)?	0.68
% total variance explained	60.1
Eigenvalue	6.39

### Convergent validity

We examined the correlation between each criterion as scored present/absent on the BPD module of the SIDP-IV and the MSI-BPD. We found that all rho coefficients were highly significant (*p* < 0.001) and that they ranged from a high coefficient of 0.86 (Q2) to a low coefficient of 0.45 (Q6).

### Criterion validity

**Fig. 2:**
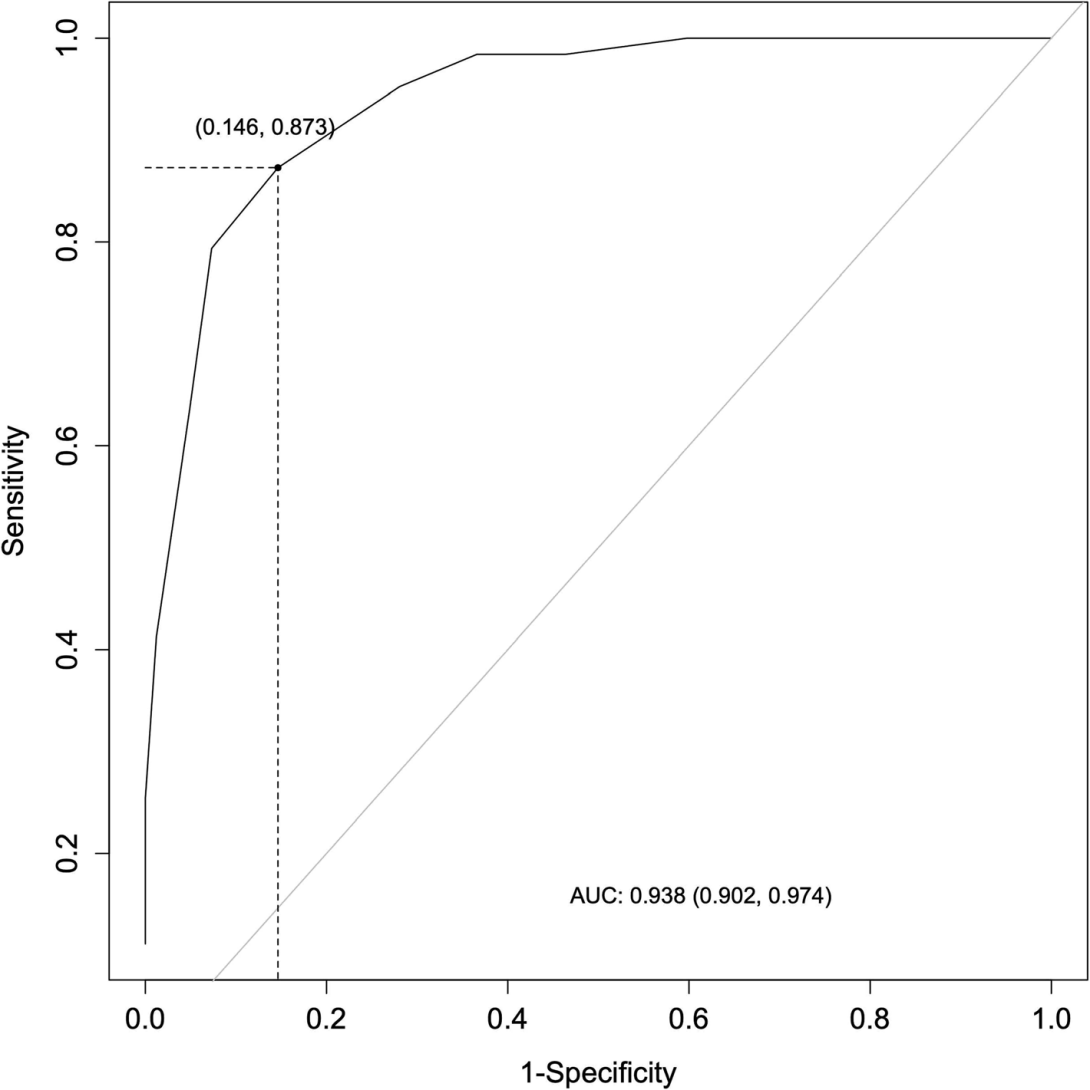
Diagnostic efficiency of the MSI-BDP receiver operating characteristics (ROC) analysis

Analysis of the receiver operating characteristics (ROC) demonstrated that the MSI-BPD had high effectiveness as a screening tool: area under the ROC curve (AUC) = 0.93, CI 95%: 0.90–0.97. Using the ROC analysis to evaluate sensitivity, specificity, and the positive and negative likelihood ratios of all the possible cut-off points, we determined that 5 was the optimal cut-off point, as it had relatively high sensitivity (0.87) and specificity (0.85) (Fig. 2 and Table 2). In our analyses, the cut-off point of 7, previously established as optimal, shows lower sensitivity (0.63) and high specificity (0.95).

**Table 2:** Cutoff Points and Diagnostic Efficiency

Cutoff Points and Diagnostic Efficiency	Sensitivity	Specificity	PLR	NLR
Cutoff Point				
≥ 5 (Present sudy)	0.87	0.85	5.97	0.15
≥ 6	0.79	0.93	11.29	0.2
≥ 7 (Zanarini et al., 2003)	0.71	0.96	18.44	0.30

*PLR* positive likelihood ratio, *NLR* negative likelihood ratio

## Discussion

This study examined the diagnostic value of the French MSI-BPD in a clinical borderline sample and a non-clinical control sample. To our knowledge, this is the first study to assess the MSI-BPD in a population of borderline adolescents. We found that the MSI-BPD demonstrated high diagnostic efficiency (AUC = 0.93), with a cut-off of 5 showing a good correlation with the SIDP IV (sensitivity = 0.87; specificity = 0.85). The sensitivity of our study was among the highest of the available studies and specificity was close to that originally reported by Zanarini and colleagues [5]. Our results are slightly different to those reported in an adolescent and young adult sample by Chanen and colleagues (sensitivity = 0.68, specificity = 0.75, AUC = 0.73), and in an adolescent inpatient population by Noblin and colleagues [12] (sensitivity = 0.71, specificity = 0.65, AUC = 0.73).

We found that a cut-off score of 5 increased the diagnostic efficiency of the MSI-BPD, with particularly improved sensitivity. It should be noted that the cut-off threshold established in the current study is lower than the scores established in the initial validation study [5] (≥7; Zanarini and colleagues) and in several subsequent studies [13, 15, 17, 19]. Our optimal cut-off is below the initially defined threshold of 7 [5], but it’s close to a score of 5.5 which was found by Noblin and colleagues [12] in a sample of adolescent inpatients. Several hypotheses may be proposed to explain our results, which are significantly different from other studies using similar samples, i.e. adolescents and young adults [12–14]. Firstly, the sample sizes of the available studies were smaller overall, ranging from 16 [14] to 31 [12]. Secondly, in the three studies cited above, the control groups were made up of adolescents who were not borderline but who were receiving mental health care whereas our control group was composed of normally developing adolescents. Finally, the severity level of the psychopathology varies considerably because some samples are composed of ambulatory [13, 14] and others of hospitalized subjects [12].

The cut-off score recommended to distinguish “cases” from “non-cases” in a questionnaire with a continuous score distribution should depend on the intended use of the scale. If the objective is to identify a relatively homogeneous group of individuals who are very likely to have the disorder being investigated, then a high threshold will be chosen to increase the specificity of the scale and thus reduce the number of false positives. If, however, the objective is a broad screening, the threshold chosen must be lower to increase sensitivity.

The current study has several limitations. Since MSI-BPD is a self-questionnaire, several biases may be present such as the bias of social desirability or social conformity. Subjects with co-morbid Axis I psychiatric disorder were not excluded, nor were subjects who were receiving psychotropic drugs. More specifically, our sample included 37% of adolescents with a major depressive episode. The clinical distinction between depressive disorders and BPD is not easy to make, especially in adolescents, because numerous symptoms overlap [24]. It would have been appropriate to have a group of adolescents with BPD but without depressive disorder, in order to have a clearer idea of the value of the MSI BPD in cases of associated depression. Unfortunately, the size of our sample did not allow us to carry out such analyses. This limitation should be taken into account for designing future studies.

The cut-off score presented in the current study should be interpreted and used with caution due to these limitations of sampling variability.

## Conclusion

In conclusion, this study adds to the growing number of studies suggesting that the MSI-BPD appears to be a feasible screening tool for BPD. Although screening does not replace the use of semi-structured interviews, the MSI-BPD can help to screen borderline subjects and ultimately reduce diagnostic delay.

## Data Availability

The datasets used and analysed during the current study are available from the corresponding author on reasonable request.

## Abbreviations

BPD: Borderline Personality Disorder
MSI-BPD: McLean Screening Instrument for Borderline Personality Disorder
SIDP-IV: Structured Interview for DSM-IV Personality
DIPD-IV: Diagnostic Interview for DSM-IV Personality Disorders
